# Acute Diffuse Nephritis

**Published:** 1884-02

**Authors:** Francis Delafield

**Affiliations:** Professor of Pathology and Practical Medicine, College of Physicians and Surgeons


### Acute Diffuse Nephritis.

By Francis Delafield, M. D.,

Professor of Pathology and Practical Medicine, College of Physicians and Surgeons.

Gentlemen—I was speaking to you yesterday of acute diffuse nephritis, and I called your attention to the fact that there were two different classes of cases characterized by different clinical histories. I told you that in the first group of cases the invasion of the disease was usually acute and the symptoms were developed rapidly, and at the same time there was a marked febrile movement.

We now come to the second group of cases of acute diffuse nephritis. In this set the invasion of the disease is not usually acute and well marked, but it is slow and gradual. There are no rigors and no febrile movement, and very often the patient cannot tell you the precise day on which the symptoms commenced, for they have begun slowly and gradually, and have increased steadily. In most cases the patients will tell you that they suddenly found they were beginning to lose their appetites and to be troubled with nausea, and some of them with vomiting also, though this is not a constant symptom. They then began to have pain in the back, of moderate intensity, and referred to the lumbar region. This, however, is always a very unreliable symptom, for perhaps half of all patients suffering from any of the ordinary diseases will tell you at some time that they have pain in the back. It is a very common symptom indeed. These patients find also that there is a loss of bodily strength and muscular force, and an indisposition to either muscular or mental exertion, and they do not feel in their ordinary mental or bodily condition. Then they notice, sometimes, at first, or in some cases not until after some time, that their urine is beginning to be diminished in amount, but this diminution does not usually reach any great degree. The patients pass, perhaps, twelve, or fourteen, or sixteen ounces in the twenty-four hours. This urine may be natural in color and appearance, or it may be changed somewhat by the addition of a certain amount of blood. When we come to examine the urine we find that its specific gravity is low, although it is diminished in amount. Albumen is present in large amount, and we also find all sorts of casts present in very considerable numbers.

Dropsy is developed very early in the disease, and it regularly reaches a very considerable degree. If it is confined to the legs, they will become very much swollen, or it may involve the subcutaneous connective tissue throughout the body, and any of the serous cavities besides. The dropsy, then, is the most marked symptom in this class of cases.

Cerebral symptoms are generally almost altogether absent, or they reach no great degree of development. There may be some dyspnœa, but this is not excessive. The color of the patient changes, and the skin and mucous membranes become pale, and this is accompanied by a change in the composition of the blood.

These symptoms may all be developed during the course of a few weeks, and after this they last sometimes for weeks, and sometimes for several months. Then, after this condition has persisted for a number of weeks or months, there may be a gradual improvement in the condition of the patient; the appetite returns, the dropsy diminishes in amount, the urine increases in quantity, the albumen becomes less marked, the casts are less and less numerous, and the patient continues to improve until the dropsy has disappeared and he is able to be out and to go about again. But the anæmia does not disappear at once, but continues for some time after, and there will also be still a moderate amount of albumen and a few casts in the urine. Then, after a still longer time, these symptoms disappear also, and the patient is restored to his natural health.

But in other cases the course of the disease is not so favorable, for it continues to advance and becomes chronic, and then the patients go on and have the symptoms and lesions of chronic diffuse nephritis. This continuance of the disease is often separated from the more acute stage by a sort of interval. That is, the patients first have the ordinary symptoms of acute diffuse nephritis of this particular variety, then, after a time, they get better, the dropsy disappears, and they only complain of feeling a little weak. But some albumen and a few casts usually remain in the urine. In other cases there seems to be an absolute recovery. This freedom from symptoms lasts for several months, perhaps, then the old symptoms come back again, the dropsy, the disturbances of the stomach, and the anæmia, all return and do not disappear again, but go on getting constantly worse and worse, until the patient finally dies at the end of several months. On examining the kidneys of such a patient, you do not find the changes of a simple form of acute diffuse nephritis, but they look rather as if they had originally been the seat of an acute diffuse nephritis which had afterward become chronic. So, after an apparent recovery, you must not be in too much of a hurry to dismiss these patients from your observation, for the symptoms may return after they have been absent for a number of months. Most of the examples of this form terminate in one of two ways—either they end in recovery, or else they pass on into the chronic condition.

The treatment of this group of cases is mainly directed toward diminishing the dropsy, and we may attempt to do this in the same way as in cases of acute parenchymatous nephritis. We may try the effects of cathartics and diuretics, and of sweating the patient, and see if by these means the amount of urine is increased and the dropsy diminished. If no effect is produced within a few days, then stop the treatment and wait a while, then try again, and, when the proper time has arrived, the remedies will act, the urine will increase, and the dropsy diminish or disappear. Then we have to attend to feeding the patient and to improving the condition of the blood in practically the same way as in acute parenchymatous nephritis. You will observe I have presented to you the symptoms of this set of cases of acute diffuse nephritis as the same as the symptoms of acute parenchymatous nephritis, and it must be confessed that in a large number of cases, during life, we cannot distinguish between these two diseases, for the symptoms are the same. Therefore the treatment is the same, and there is very much the same prognosis.

A considerable number of cases of acute diffuse nephritis occur in the course of scarlet fever, and thus become a complication of it. But you must remember that, with scarlet fever, acute parenchymatous nephritis is also a common complication. So when you use the term "scarlatinal nephritis," you must do so remembering that there are these two forms of nephritis which may complicate a scarlet fever, an acute parenchymatous and an acute diffuse inflammation of the kidneys. There are apparently but very few cases of scarlet fever that run their course without the presence of either one or the other of these two forms of renal disease as a complication, and acute parenchymatous nephritis is the more common one, while the acute diffuse inflammation is the less common. There are also some cases of scarlet fever that run their regular course throughout, and you would never suppose that there was any accompanying disease of the kidneys if you did not examine the urine. But yet, when you do examine it, you find some albumen and a few casts, which are evidences of a parenchymatous inflammation. When, however, a diffuse nephritis is present, it does give symptoms, and there are not only changes in the urine, but also constitutional symptoms, varying in severity in different cases.

The precise time in the course of the scarlet fever at which the kidney lesion is developed varies a good deal in different cases, and we are unable to determine this just as we are unable to diagnosticate between cases of parenchymatous and diffuse nephritis. The larger number of these renal complications do not result in the death of the patient, and, as only a moderate number die, we cannot determine very often the exact nature of the renal disease. Taking these two sets of cases together, however, it is found that the renal symptoms may make their appearance at any period between the first day of the scarlet fever and the ninth week after the invasion. But the great majority of cases occur on or about the fourteenth day of the scarlet fever, and next in frequency comes the twenty-first day, and next the seventh day. So, if we take all the cases of scarlet fever complicated with renal disease together, in most of them we shall find that the kidney complication is developed either at the fourteenth, the twenty-first, or the seventh day, and the most frequent of these is about the fourteenth day.

These patients give us first the ordinary symptoms of scarlet fever, sometimes severe in form and sometimes mild; for this complication seems to be as apt to occur with mild as with the severe cases of scarlet fever; and then, after the ordinary symptoms of the scarlet fever have lasted it may be for one, two, or three weeks or more, there is noticed a change in the condition of the patient—he is evidently worse, and a loss of appetite makes itself evident. If the renal disease is not developed until the fourteenth or twenty-first day, then very likely the patient will be feeling better of the scarlet fever, when suddenly rigors appear, and then, if the appetite has returned with the improvement in the scarlet fever, it will now be lost again, and there may be added more or less nausea and perhaps vomiting. In many cases there will, at this time, be developed a new febrile movement, and, if the fever of the scarlet fever is still continuing, it becomes higher than before, while if it has already reached the normal there will now be an increase of temperature, but the new febrile movement will not be of any great degree of severity. The patients will now begin to complain of pain in the back, and they will become fretful and restless and be unable to sleep well at night, and between these periods of restlessness and sleeplessness they will be unnaturally dull and stupid, and, if it is a grown-up person or a pretty old child, he will complain of headache. In bad cases there may be developed delirium, convulsions, and a very marked stupor, which may go on to complete unconsciousness.

Dropsy is developed, sometimes only to a very moderate extent, and sometimes there is very marked general anasarca. There are the ordinary changes in the blood, and the patient's appearance becomes anæmic. The urine is scanty or absolutely suppressed; it has a low specific gravity and contains some blood, and albumen is present in large amount, and there are numerous casts.

These symptoms, when once developed, last, in most cases, about two weeks, then all the symptoms begin to diminish, and finally they disappear altogether. The changes in the urine, however, continue for a considerable time after the patient has apparently entirely recovered.

The prognosis in most of these cases of nephritis complicating scarlet fever is good, and the patients get better usually after the second week. But sometimes the renal complication will be more severe in character, and prove fatal either at the end of a few days, or perhaps at the end of two or three weeks. Then in still other cases the acute kidney lesion changes into a chronic lesion, and it goes on and develops into a chronic diffuse nephritis.

As regards the treatment of these cases of scarlatinal nephritis, we find that some of them hardly require any treatment at all. The urine is not very much diminished and the symptoms are not very intense, and all that is necessary is to keep the child in bed and keep the bowels open with some simple laxative, and give it only a fluid diet, and then wait quietly for the symptoms to subside. But, in case the symptoms are more severe, it may be necessary to interfere, and we may apply cups or hot fomentations over the lumbar region, or sweat the patient and give more vigorous cathartics, and in some cases it is also necessary to give opium to quiet the extreme restlessness and sleeplessness. Then, when the symptoms subside, if the patient is pretty well nourished, no further treatment is necessary, but there may be a gradual return to animal food, and the opium may be withheld. But, if the patient is not in good condition, then you must look after the general nutrition, and try to improve the condition of the blood in the ordinary way.—*N. Y. Md. Journal.*

